# Microglia, Cytokines, and Neural Activity: Unexpected Interactions in Brain Development and Function

**DOI:** 10.3389/fimmu.2021.703527

**Published:** 2021-07-01

**Authors:** Austin Ferro, Yohan S. S. Auguste, Lucas Cheadle

**Affiliations:** Neuroscience Department, Cold Spring Harbor Laboratory, Cold Spring Harbor, NY, United States

**Keywords:** brain, synapse, microglia, cytokine, neural activity, sensory experience, development

## Abstract

Intercellular signaling molecules such as cytokines and their receptors enable immune cells to communicate with one another and their surrounding microenvironments. Emerging evidence suggests that the same signaling pathways that regulate inflammatory responses to injury and disease outside of the brain also play powerful roles in brain development, plasticity, and function. These observations raise the question of how the same signaling molecules can play such distinct roles in peripheral tissues compared to the central nervous system, a system previously thought to be largely protected from inflammatory signaling. Here, we review evidence that the specialized roles of immune signaling molecules such as cytokines in the brain are to a large extent shaped by neural activity, a key feature of the brain that reflects active communication between neurons at synapses. We discuss the known mechanisms through which microglia, the resident immune cells of the brain, respond to increases and decreases in activity by engaging classical inflammatory signaling cascades to assemble, remodel, and eliminate synapses across the lifespan. We integrate evidence from (1) *in vivo* imaging studies of microglia-neuron interactions, (2) developmental studies across multiple neural circuits, and (3) molecular studies of activity-dependent gene expression in microglia and neurons to highlight the specific roles of activity in defining immune pathway function in the brain. Given that the repurposing of signaling pathways across different tissues may be an important evolutionary strategy to overcome the limited size of the genome, understanding how cytokine function is established and maintained in the brain could lead to key insights into neurological health and disease.

## Introduction

The secretion of soluble signaling proteins by immune cells (e.g. macrophages, circulating monocytes, and lymphocytes) and the activation of their cell-surface receptors via ligand binding regulate many important processes throughout the body. Over the past 50 years, immunologists have profiled a vast array of immune signaling pathways (broadly referred to as cytokines and cytokine receptors) that mediate distinct functions in a highly context-dependent and tissue-specific manner. This large family of signaling pathways, broadly defined, includes tumor necrosis factors (TNFs), interferons (IFNs), chemokines, colony stimulating factors (CSFs), interleukins (ILs), complement proteins, major histocompatibility complex proteins (MHCs), and their many and varied receptors. These pathways allow immune cells to shape a host of critical processes within target tissues, including cell proliferation, cell migration, angiogenesis, inflammation, and tissue damage and repair (1). Although many of these functions involve core mechanisms shared across biological systems, the downstream molecular changes and functional outcomes elicited by cytokine signaling pathways can also vary significantly from tissue to tissue.

The functional adaptability of cytokines to different contexts may represent a critical biological strategy to overcome the size limits of the genome through the molecular repurposing of a defined number of signaling molecules. Nowhere is the need for a rich repertoire of specialized signaling mechanisms more apparent than in the central nervous system (CNS), comprised of the brain and spinal cord. Despite making up only ~2% of total body weight, the brain uses ~20% of the body's energy, consistent with the brain being constantly engaged in highly active functions (2–4). Moreover, the precise establishment of the brain's complex anatomy during development, including cellular organization and synaptic connectivity, requires a host of signaling mechanisms specific to age, cell type, and subregion of the brain. Although signaling networks such as ephrins and semaphorins are known to coordinate many aspects of neural development and circuit wiring (5, 6), the vast complexity of the brain suggests that these pathways represent a slim fraction of signals used when it comes to molecular mechanisms underlying neurological form and function. This observation underscores the need for a systematic characterization of the myriad signaling pathways that organize the brain.

While the roles of cytokines outside of the brain have been studied for decades, their functions in the healthy brain have been unexplored until relatively recently. This is in large part due to a long and widely held understanding of the brain as an "immunologically privileged" site in which the neurovasculature, surrounded by a specialized cellular scaffold called the blood-brain barrier (BBB), is largely impenetrable not only to circulating immune cells but to soluble cytokines as well (7). This classical view has shifted over the past ~15 years thanks in large part to two discoveries: (1) canonical pro-inflammatory cytokines such as TNFα, IFNγ, and IL-1β regulate synaptic transmission between neurons (8, 9); [reviewed (10, 11)], and (2) the resident immune cells and predominant cytokine expressers of the CNS, microglia, play an astoundingly wide array of previously unappreciated roles in the healthy brain (12, 13). Yet, it remains to be determined how microglia and cytokines play such specialized roles in the development and function of the CNS compared to their roles in the context of immunity.

Tissue-specific signals are likely to control at least some aspects of cytokine function, and one particular signal that is largely unique to the CNS is neural activity. When a neuron receives information from another neuron in the form of chemical neurotransmission at the synapse (i.e. the presynaptic release of a neurotransmitter onto specialized receptors in the postsynaptic membrane), this chemical signal is transduced into an electrical signal. The electrical signal then propagates across the neuron's membrane and ultimately results in the subsequent release of neurotransmitter onto downstream neurons, leading to the electro-chemical information flow of the brain. *Neural activity* is a broad term encompassing the propagation of these electro-chemical impulses across and between individual and/or networks of neurons, and changes in neural activity can alter the amount of neurotransmitter released at the synapse as well as the conformation, ion conductance, and density of neurotransmitter receptors in the postsynaptic membrane. Other consequences of neural activity include the activation of dynamic calcium (Ca^2+^ (fluctuations that signal from the synapse to the nucleus to coordinate stereotyped gene programs encoding critical molecular regulators of synaptic connectivity and function (14). Therefore, electrical activity and downstream neurotransmitter release are associated with defined molecular and cellular changes.

As the primary means of information transfer across neural circuits, activity regulates virtually all aspects of neurological function. Therefore, activity is a promising candidate factor to shape microglial and cytokine function in the brain. Indeed, emerging evidence suggests that microglia and cytokines not only participate in neurological processes that are powerfully controlled by activity, but that the roles of microglia and cytokines in the brain *rely upon* their ability to respond to changes in activity. Here, we review the most recent evidence that neural activity coordinates microglial and cytokine function in the brain. We also discuss the implications of these activity-dependent, cytokine-mediated processes for neurological function, describe the aspects of these processes that remain under active investigation, and discuss the most pressing questions that have yet to be addressed about the roles of immune signaling pathways in the CNS.

## Microglial Function Is Shaped by Activity

Unlike neurons and other brain cells, microglia originate from an erythromyeloid progenitor pool in the yolk sac, migrate into the embryonic brain *before* the BBB is formed, and protect the brain from injury and disease by clearing damaged tissue through phagocytic engulfment (12). While microglia are not the only cells in the brain that express cytokines and cytokine receptors, mounting evidence reveals that they engage these pathways to shape a host of critical and specialized brain functions including neuronal apoptosis, synapse formation, synapse elimination, circuit homeostasis, circuit plasticity, and complex behaviors (15–20). Understanding how these signaling pathways that coordinate inflammation in the periphery are repurposed to perform highly specialized, non-injury induced functions in the brain is key to establishing a systematic understanding of the molecular basis of brain function. Moreover, based upon evidence that immune dysfunction contributes to a host of neurological disorders emerging across the lifespan (discussed in more detail later), elucidating these mechanisms may facilitate novel therapeutic strategies for treating human disease by uncovering promising diagnostic biomarkers and drug targets.

### Microglial Responses to the Artificial Manipulation of Activity

*In vivo* imaging of brain tissue in living mice via two-photon microscopy is a rapidly evolving approach in neuroscience that has been valuable in characterizing the physiological responses of individual brain cells to diverse stimuli. In particular, functional imaging of Ca^2+^ dynamics using genetically encoded Ca^2+^ sensors, such as Gcamp6, provides a readout of cellular activation and has been extensively applied to neurons and astrocytes, a non-neuronal brain cell type that is tightly attuned to neural activity (21). Yet, until recently these Ca^2+^ imaging strategies had not been applied to microglia. In a groundbreaking study, Umpierre et al. (22) performed functional Ca^2+^ imaging in microglia of the somatosensory cortex in live, unanesthetized mice. They discovered that changes in neural activity initiated by exposure of the mice to kainate, a seizure-inducing drug that drastically increases activity, led to changes in Ca^2+^ dynamics within the highly ramified and motile processes of microglia. More selective bidirectional manipulation of neural activity through the exogenous expression of either excitatory or inhibitory Designer Receptors Exclusively Activated by Designer Drugs (DREADDs), molecular tools for precisely manipulating neural activity (23), in nearby neurons also produced tightly attuned transient changes in Ca^2+^ dynamics within microglial processes. Remarkably, Ca^2+^ dynamics localized to microglial processes were induced within minutes of either an acute decrease *or* increase in the activity of neighboring neurons, and the patterning of these activity-induced Ca^2+^ fluctuations was strikingly similar following either an increase or a decrease in activity. These results are consistent with a role for microglia in carefully monitoring activity at nearby synapses to maintain circuit homeostasis, i.e. the balance of excitation and inhibition present within a given circuit (22).

What impact does the functional coupling of microglial Ca^2+^ dynamics to neural activity have on the wiring of the brain? While this is still being investigated, a number of structural live-imaging studies lend support to the idea that microglia regulate synaptic connectivity in an activity-dependent manner. For example, in a resting state, microglia actively survey the parenchyma of the cerebral cortex and directly contact synapses about once per hour. Initially, cortical microglia were shown to decrease synaptic contact frequency upon the dampening of neural activity by local application of the activity blocker tetrodotoxin (TTX) (20). Yet, this early finding has recently been contradicted by more refined manipulations of neural activity. For example, inhibiting activity through the injection of the inhibitory neurotransmitter Gamma Aminobutyric Acid (GABA)-agonist muscimol, suppression of activity via optogenetic stimulation of inhibitory neurons, or lowering overall neural activity using general anesthetics were shown to increase microglial process surveillance and motility in the somatosensory cortex (24). Consistent with this result, fixed tissue studies of the retinogeniculate circuit, a pathway that connects retinal ganglion cells (RGCs) in the eyes to the dorsal lateral geniculate nucleus (dLGN) of the thalamus in the brain, showed that microglia are more likely to phagocytically engulf synapses that have been silenced by TTX injection into the retina as compared to unsilenced, active inputs (17). These data suggest that decreasing activity can have versatile effects on microglia but in most cases leads to increased contact between microglia and synapses.

**Figure 1:**
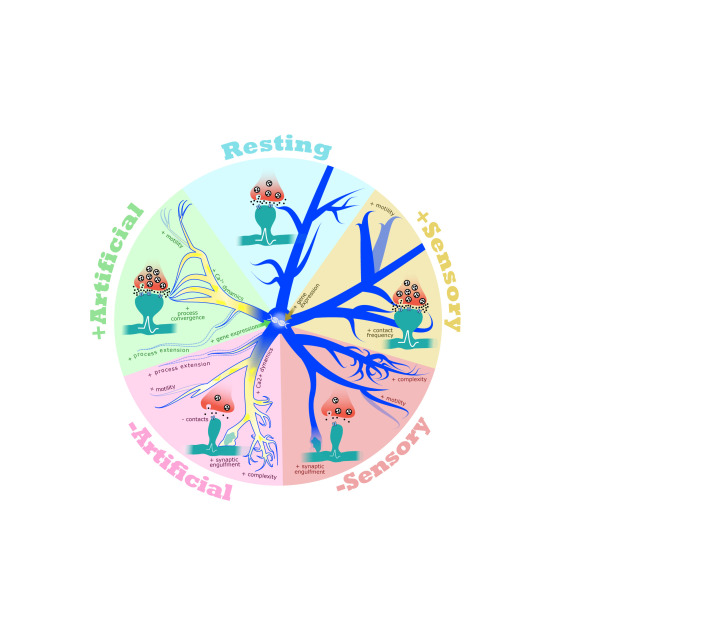
Microglia respond to changes in neural activity. In a resting state, microglia (blue) actively survey the neural environment, dynamically contacting synapses (presynaptic terminals in red, postsynaptic dendrites and spines in teal). Artificial manipulation of neural activity by pharmacological, chemogenetic, or optogenetic perturbation leads to robust changes in microglial calcium dynamics (shown in yellow), process motility, synaptic contact frequency, and transcription. Depriving mice of or exposing mice to sensory stimuli affect many of the same aspects of microglial function. These data are consistent with an important role for microglia in establishing and/or maintaining circuit stability and homeostasis.

In parallel with studies employing artificial methods to inhibit activity, several other studies have used pharmacological or chemogenetic tools to assess the effects of heightened activity on microglial function. For example, in the cortex, the frequency of microglia-synapse contacts increases as a result of blocking the inhibitory neurotransmitter GABA, a manipulation that heightens neural activity (18). This result is consistent with work showing that increasing neural activity using Bicuculline, an antagonist of GABA, increases microglial motility overall (18). Increasing neural activity through excitatory DREADDs also increased the frequency of microglia-synapse contacts in the striatum (25), indicating that this phenomenon is not selective to cortex. Indeed, similar findings were observed in the hippocampus, where exposure to kainate was shown to increase microglial process convergence, a phenomenon in which multiple microglial processes are simultaneously drawn toward a specific synapse or group of synapses, which likely reflects an increase in contacts between microglia and synapses (22, 26–28). Induction of seizures with kainate also induced changes in Ca^2+^ dynamics (22), active territory surveillance (26) synaptic engulfment (28), and gene expression in microglia (25, 29), highlighting that microglia respond to increases in activity by mechanisms that are largely similar to, but in some ways distinct from, the effects of dampening activity (**Figure 1**). Given that kainate raises activity enough to induce seizures, some of these effects may reflect pathological changes and should thus be interpreted carefully. Nevertheless, these findings highlight that the functional responses of microglia to neural activity are accompanied by robust structural and molecular changes. Just as neural activity drives changes in microglia, microglia also provide feedback to neurons, for example through the conversion of adenosine triphosphate into adenosine which leads to suppression of neural synchrony and firing (25). Consistent with bidirectional interactions between microglia and neurons being organized by activity, microglia are also required for learning-dependent spine turnover in motor cortex, further suggesting that microglial function is tightly linked to activity-dependent processes in the brain (30).

### Microglial Responses to the Manipulation of Sensory Experience

While pharmacological, chemogenetic, and optogenetic methods for altering neural firing have provided compelling evidence that microglia can sense and respond to activity, how microglia respond to physiologically relevant stimuli such as sensory experience is an ongoing topic of research. It is now well understood that microglia in the visual system routinely contact and eliminate synaptic elements such as presynaptic boutons and postsynaptic structures called dendritic spines (17, 20). Depriving mice of visual experience by rearing them in complete darkness (dark-rearing) leads to an increase in microglia-synapse contact frequency and an increase in the engulfment of neuronal material, as well as the expansion of microglial processes in visual cortex. Interestingly, re-exposure of mice to light following a period of dark-rearing also increased the number of microglia-synapse contacts compared to normally reared mice (31), consistent with the observation that microglial calcium signaling is similarly induced by either a decrease or an increase in activity (22). On the other hand, the motility of microglia was decreased by dark-rearing but increased by re-exposure of dark-reared mice to light, suggesting that distinct mechanisms induce microglia-synapse interactions in each case (31).

The contribution of microglia to experience-dependent developmental plasticity within the brain is not restricted to the visual cortex. For example, in the dLGN, the elimination of synapses during the third week of postnatal life, a stereotyped developmental process that requires visual experience, also requires cytokine signaling by microglia (32). Similarly, sensory experience in the somatosensory cortex, a brain region that processes tactile information, also shapes microglial function. For example, depriving adult mice of experience by whisker trimming (i.e. removing sensory information mediated by facial whiskers) leads to increased process surveillance and motility by microglia, suggesting that, when experience is dampened, microglia may spend more time and energy seeking active synapses (24). In young mice, at P4-5 when the cortical territories for each whisker are still being formed, sensory deprivation by whisker lesioning induces microglial engulfment of thalamic inputs to cortex, a process that requires fractalkine signaling from neurons to microglia and results in the functional refinement of thalamocortical connectivity (33). Therefore, in both the visual system and the somatosensory system, manipulating sensory experience leads to diverse, bidirectional changes in microglial motility and contact with synapses.

Altogether, these imaging data highlight that the roles of microglia in synapse development and plasticity are likely to be reliant upon robust microglial detection of changes in neural activity within their microenvironments. Yet, a caveat to be taken into consideration is that many of these studies were performed in mice anesthetized with either isoflurane or ketamine, drugs that suppress neural activity overall and which Umpierre et al. (22) found were sufficient to drive increases in Ca^2+^ dynamics and process extension in microglia. To enable the careful interpretation of these results, we provide annotation in **Table 1** of which live-imaging studies were performed in anesthetized versus awake mice. Despite this caveat, this expanding body of work demonstrates that many features of microglial function - e.g. Ca^2+^ dynamics, contact with synapses, and process motility – can be similarly affected by either decreased or increased activity, indicating a potential role for microglia in balancing and maintaining circuit homeostasis (**Figure 1**). This observation suggests that microglia may be just as important for synapse formation, strengthening, and maintenance as they are for synapse elimination. Further studies are required to better understand how microglia work in concert with neural activity to regulate synaptic connectivity and homeostasis, as well as to determine the contributions of microglia to more global aspects of neural circuit function beyond the local regulation of synapses.

**Table 1:** Microglia respond to changes in neural activity by altering their motility and interactions with synapses.

*Activity perturbation*	*Effect on microglia*	*Mechanism/Mediator*	*Brain region*	*Anesthetic used*	*Citation*
Decreased neural activity (general anesthesia, whisker trimming)	Increased motility, process complexity	Norepinephrine	SS (somatosensory) cortex	None	Liu et al. (24)
Decreased neural activity (TTX)	Increased synaptic engulfment	Complement pathway (C1/Cr3)	dLGN	Fixed tissue	Schafer et al. (17)
Decreased neural activity (isoflurane, Gi DREADD)	Increased calcium signaling and process extension	Unknown	SS cortex	None	Umpierre et al. (22)
Decreased neural activity (TTX, decreased body temp.)	Decreased synaptic contacts	Unknown	Visual cortex	Ketamine and Xylazine	Wake et al. (20)
Decreased neural activity (light deprivation)	Decreased motility	Unknown	Visual cortex	Fentanyl, Midazolam, and Medetomidine	Tremblay et al. (31)
Decreased neural activity (whisker lesioning)	Increased engulfment	Adam10/Fractalkine	SS cortex	Fixed Tissue	Gunner et al (33)
Increased neural activity (glutamate, kainate)	Increased process extension	P2Y12	Hippocampus (*Ex vivo*) and SS cortex (*in vivo*)	Unknown	Eyo et al. (27)
Increased neural activity (pilocarpine, kainate)	Increased microglial process convergence	Cx3cr1	Cortex	Isoflurane	Eyo et al. (28)
Increased neural activity (kainate, Gq DREADD)	Increased calcium signaling and process extension	Unknown	SS cortex	None	Umpierre et al. (22)
Increased neural activity (bicuculline)	Increased motility	Unknown	SS cortex	Ketamine and Xylazine/Isoflurane	Nimmerjahn et al. (18)
Increased neural activity (Gq DREADD)	Microglial negative feedback to neural activity	ADP release from microglia	Striatum	None	Badimon et al. (25)
Increased neural activity (Gq DREADD) or ischemia	Increased somatic contact	P2Y12	Cortex	Fentanyl	Cserép et al. (34)
Increased neural activity (kainate)	Change in gene expression	Unknown	Hippocampus	N/A	Bosco et al. (29)
Increased neural activity (light stimulation)	Increased contact frequency and process motility	Unknown	Cortex, V1	Fentanyl, Midazolam, and Medetomidine	Tremblay et al. (31)
Neural NMDAR activation (NMDA)	Increased motility	NMDA-based release of ATP	Cortex	Fentanyl, Midazolam, and Medetomidine	Dissing-Olesen et al. (26)
Tetrapentylammonium (TPA), Increased [K^+^_o_]	Decreased neural surveillance	THIK-1	Cortex	Urethane	Madry et al. (18)

## Cytokine Signaling in Neurodevelopmental Processes Shaped by Activity

A body of live-imaging and fixed tissue studies demonstrate that microglia function in concert with neural activity to shape brain wiring. While the molecular mechanisms governing this process have not yet been extensively defined, microglia are derived from an immune cell lineage which makes canonical immune signaling molecules appealing candidates to link microglia to activity-dependent changes at synapses. Furthermore, evidence suggests that immune signaling molecules in the brain are not exclusively expressed by microglia, indicating that non-immune cells like neurons and other non-neuronal glial cells could also engage these powerful molecular pathways to drive neurological function. Below, we review evidence that intercellular signaling pathways associated with immune responses in the periphery are repurposed to orchestrate many aspects of neural development and plasticity in a manner that depends upon age, circuit, and pattern of activity.

### The Classical Complement Cascade

The roles of immune signaling molecules in brain development have been extensively studied in the retinogeniculate circuit of the mouse. This connection between the axons of RGCs in the eye and their thalamic postsynaptic targets, the relay neurons of the dLGN, undergoes a robust period of circuit refinement spanning the first month of postnatal life that involves a progressive decrease in the number of retinogeniculate synapses and a concurrent increase in the strength of the remaining connections (35–38). Retinogeniculate refinement can be broken down into separate activity-dependent phases which are mediated by distinct immune signaling pathways, beginning with eye-specific segregation (ESS) that starts at birth and culminates around P7 in mice. During this phase, inputs deriving from RGCs in the ipsilateral and contralateral eyes that synapse onto the same territory in the dLGN are selectively eliminated, leading to the anatomical segregation of ipsi- and contralateral synapses. Terminating prior to the onset of visual experience at eye-opening, ESS is driven by intrinsically generated neural activity propagated to the dLGN from the retina (39). The first evidence that this early stage of refinement in the dLGN involves immune signaling mechanisms emerged in 1998 from an elegant study from the lab of Dr. Carla Shatz which showed that class 1 MHC proteins are expressed in dLGN neurons in an activity-dependent manner and are required for ESS (40). This and subsequent related studies on the roles of MHC molecules in synaptic refinement (41–43) opened a new line of investigation into interactions between immune pathways and developing synapses, paving the way for Drs. Ben Barres and Beth Stevens to discover key roles for the classical complement cascade (ccc) in retinogeniculate refinement.

The ccc is an innate immune pathway that mediates phagocytosis in peripheral tissues and in the brain. In the CNS, microglia initiate the cascade by releasing the secreted molecule complement component 1q (C1q), which binds dead cells, debris, or even synapses and triggers the synaptic deposition of the downstream complement component 3 (C3). C3 deposited on synapses is recognized by the CR3 receptor expressed by microglia which induces microglia to phagocytose the complement-tagged synaptic material thereby disassembling functional synapses.

**Figure 2:**
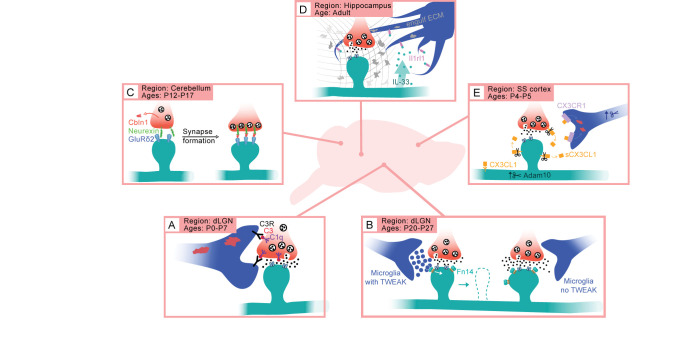
Cytokine pathways coordinate brain development and plasticity in a region- and age-dependent manner. **(A)** In the dLGN of the thalamus, the classical complement cascade (ccc) mediates the phagocytic engulfment of synapses (presynaptic inputs in red, postsynaptic dendrites and spines in teal) by microglia (blue) between birth and P7. **(B)** During a later period of experience-dependent refinement in the dLGN, TWEAK-Fn14 signaling from microglia to neurons disassembles synapses, while Fn14 functions in the absence of its ligand TWEAK to strengthen synaptic connections. **(C)** In the cerebellum, the cytokine Cbln1 binds presynaptic Neurexin and the postsynaptic neurotransmitter receptor GluRδ2 to establish parallel fiber – to – Purkinje cell synapses independently of microglia. **(D)** IL-33 to Interleukin 1 receptor-like 1 signaling mediates experience-dependent synaptic plasticity in the hippocampus by inducing microglia to engulf the extracellular matrix. **(E)** Fractalkine signaling from neurons to microglia establishes somatosensory whisker territories via microglial engulfment of thalamocortical inputs.

Roles for the ccc in synapse elimination have been best demonstrated in the retinogeniculate circuit, where the ccc is critical for ESS and the continuing refinement of retinogeniculate connectivity. Originally identified in a screen for neuronal genes expressed in cultured RGCs upon exposure to astrocyte-derived media, C1q was not only shown to be expressed in the retina but also in the dLGN where it localizes to developing retinogeniculate synapses in wild-type (WT) mice. ESS is disrupted in mice lacking C1q or C3 such that ipsi- and contralateral retinal inputs are not appropriately segregated in the knockout (KO) mice. In addition to impairments in ESS, C1q KO mice also exhibit electrophysiological refinement deficits that persist at least until P30 when the circuit is largely mature, resulting in the retinogeniculate circuits of C1q KO mice maintaining an excess of weak synapses compared to wild type counterparts (44). Although initially identified in cultured RGCs, C1q and C3 are enriched in microglia *in vivo*, suggesting a role for these immune cells in eliminating developing synapses through phagocytic engulfment. Indeed, microglia from mice lacking C1q, C3, or CR3 phagocytose less synaptic material than their WT counterparts, indicating that the ccc drives synapse elimination by triggering synaptic engulfment by microglia, consistent with the nature of microglia as specialized myeloid cells (**Figure 2A**) (17). Interestingly, regulation of ESS by the ccc also requires Transforming Growth Factor beta (TGFβ) signaling in the retina, highlighting that cross-talk between distinct immune signaling axes is perhaps an additional mechanism through which cytokines perform distinct functions dependent upon context (45). Together, these studies demonstrated novel roles for the ccc and microglia in developmental synapse elimination.

Following up on these studies in the dLGN, the ccc has also been shown to regulate synaptic connectivity in cortical slices (46), and to promote developmental synapse elimination in the ventral thalamus (47) and the spinal cord (48). Moreover, ccc function is not restricted to development but has also been strongly linked to age-dependent synaptic changes in the hippocampus, a widely studied brain region associated with learning and memory (19, 47, 49). Loss of C1q leads to synaptic deficits in hippocampal neurotransmission and cognitive and behavioral impairments linked to hippocampal function in adult mice (47). Supporting evidence for the role of C1q in adult hippocampal function was found by Wang et al. (19), who show that microglia mediate hippocampal synapse loss, allowing mice to forget features of their environment that are not critical to their survival. Moreover, a growing body of literature implicates C1q as an early driver of synapse loss in neurodegeneration and other inflammatory brain conditions affecting the hippocampus (50–53)[reviewed in (54)]. Despite the multiple processes known to be regulated by the ccc in the brain, the pathway is not universal but rather functions in a circuit-specific manner. For example, the ccc is dispensable for the microglia-driven development of the whisker barrels in somatosensory cortex (33), sensory experience-dependent ocular dominance plasticity in visual cortex (55), and synapse elimination during a late phase of experience-dependent refinement in the dLGN that takes place between P20 and P30 (32)(**Figure 2B**). These data suggest that the ccc is not a ubiquitous regulator of synapse elimination but rather that the ccc pathway plays precise roles at synapses across a subset of circuits depending upon the age of an organism. This leaves room for other immune signaling pathways to join the ccc as critical regulators of brain development and function.

### Cerebellins and C1q-Like Proteins

One brain region in which synaptic refinement is not thought to require the ccc or microglia is the cerebellum, a posterior brain structure important for motor coordination among many other functions. Purkinje cells (PCs), a population of GABAergic neurons that are the major output of the cerebellar cortex, receive synaptic input from two main excitatory sources: climbing fibers (CFs) emerging from neurons in the inferior olive of the brain stem, and parallel fibers (PFs) emerging from granule cells (GCs) whose somata reside in the granule cell layer of the cerebellum. Like the retinogeniculate pathway, the Parallel Fiber-to-Purkinje Cell (PF-to-PC) and the Climbing Fiber-to-Purkinje Cell (CF-to-PC) pathways consist of stereotyped innervation patterns that emerge during development through activity-dependent mechanisms (56). Hence, the cerebellum joins the dLGN as a powerful model system in which to study activity-dependent aspects of neural circuit development.

Like the retinogeniculate circuit, the refinement of the CF-to-PC connection entails the removal of a large number of synapses and the maintenance of only a few innervations. Initially, multiple CFs provide relatively weak innervation to the soma of each PC. Between P3 and P7, only one of these inputs is strengthened at which point its synaptic terminals translocate from the soma to the proximal dendrites of the postsynaptic PC. Subsequent to the translocation of the strengthened input, the weak CF inputs remaining on the soma are eliminated across an early stage (P7–P11) and a late stage (P12-P17), leaving only a single CF innervation to each PC. Furthermore, the late stage elimination requires the normal development and establishment of PF-to-PC synapses onto distal PC dendrites (57). The C1q-like 1 (C1ql1) cytokine, which derives its name from its structural similarity to complement proteins, acts directly at CF-to-PC synapses to promote single CF strengthening and to drive the elimination of the CF inputs that fail to strengthen. C1ql1 expressed by presynaptic climbing fibers enforces this one-to-one innervation pattern between a CF and a PC by binding to the PC-expressed receptor Brain-specific angiogenesis inhibitor 3 (Bai3) that localizes to postsynaptic sites (58). These results underscore that cytokines can be employed to shape brain development through neural-specific mechanisms that don't directly involve microglia.

Cerebellins are another class of cytokines structurally related to both C1q and C1ql1 that play an important role in activity-dependent circuit refinement in the cerebellum. Upon its secretion from granule cells, Cerebellin-1 (Cbln1) orchestrates the assembly of PF-to-PC synapses during the third week of life. Mice lacking Cbln1 exhibit deficits in motor coordination corresponding with decreases in both the number and the strength of PF-to-PC synapses. Interestingly, the translocation of PF-to-PC synapses onto the distal dendrites of the PC is required for the development of CF-to-PC synapses, which is activity-dependent (59). Consistent with the requirement of PF-to-PC neurotransmission for CF-to-PC refinement, mice lacking Cbln1 aberrantly maintain PC innervations from multiple CFs into maturity (60). These deficits were strikingly similar to those observed upon the loss of GluRδ2, a glutamate neurotransmitter receptor subunit expressed at the postsynaptic sites of PCs during development. Indeed, the structural and functional development of PF-to-PC synapses was shown to require a direct interaction between postsynaptic GluRδ2, soluble Cbln1, and the presynaptic membrane protein Neurexin 1β (Nrx1β) (61–63). Thus, Cbln1, through binding directly to synaptic machinery, is able to control synapse formation and refinement in the cerebellum (**Figure 2C**). Cbln1, -2, and -4 are also expressed in parts of the brain other than the cerebellum and double and triple Cbln family member KO mice exhibit synaptic changes in brain regions such as the cortex and the thalamus, indicating more general roles for these immune signaling molecules in the developing brain that are yet to be described (64).

### Interleukins

Interleukins (ILs) represent another broad class of immune signaling molecules that are expressed in the brain. Two recent studies from the lab of Dr. Anna Molofsky have identified the cytokine IL-33 and its receptor Il1rl1 as important organizers of circuit development and plasticity across multiple brain regions. IL-33 was discovered as an astrocyte-derived, developmentally upregulated cytokine that binds its receptor Il1rl1 on microglia to dampen oscillatory activity in the thalamus. Constitutive KO of IL-33 in mice caused an increase in spontaneous neural firing and enhanced evoked activity in response to thalamic stimulation, suggesting an aberrant increase in the number of synapses (65). More precise deletion of IL-33 only in astrocytes led to an increased number of both excitatory and inhibitory synapses onto α-motor neurons, the primary output neurons of the sensorimotor circuit in the spinal cord. A similar effect was seen in mice constitutively lacking IL-33 or its receptor Il1rl1. In the spinal cord, deletion of IL-33 also decreased the amount of synaptic material found within the cellular boundaries of microglia, suggesting that IL-33 functions to eliminate synapses by inducing phagocytic synapse engulfment. Consistent with this result, local injection of IL-33 into either thalamus or spinal cord increased the amount of synaptic material found within microglia. Altogether, these data identify IL-33 as an astrocytic cytokine that binds its receptor Il1rl1 on microglia to decrease synapse number and strength across multiple circuits (65).

The expression pattern and role of IL-33 in thalamic and spinal cord development contrast its function in the adult hippocampus. Unlike the developing dLGN in which IL-33 is expressed in astrocytes, in the hippocampus IL-33 is derived from neurons and its neuronal expression is increased in response to environmental enrichment (EE), a physiological paradigm that results in heightened levels of neural activity (66). Loss of either IL-33 or its receptor Il1rl1 decreased spine density overall as well as the number of spines with dynamic filopodia, suggesting that IL-33-Il1rl1 signaling between active neurons and local microglia promotes EE-induced new spine formation and plasticity. Intriguingly, microglia were found to induce these synaptic changes by engulfing the extracellular matrix (ECM) component Aggrecan in an IL-33-dependent manner, as loss of IL-33 results in an inappropriate accumulation of Aggrecan at postsynaptic sites. Altogether, these data support that cytokine signaling between neurons or astrocytes and microglia is coordinated to shape synaptic connectivity in response to environmental stimuli in mice (**Figure 2D**).

### Tumor Necrosis Factors

The TNF superfamily is composed of 19 membrane-tethered or soluble ligands and 29 cell-surface receptors that mediate intercellular signaling between multiple cell types. TNF ligand-receptor binding elicits a variety of cellular processes including cell proliferation, cell migration, angiogenesis, inflammation, and apoptosis via a number of signaling cascades including NF-κB and MapK. Although the canonical family member, TNFα, has been shown to mediate synaptic strengthening and neurotransmission (8), roles for other members of this large family in the brain remain to be fully explored.

Following initial stages of refinement that rely upon spontaneous retinal activity, the retinogeniculate circuit undergoes a critical period of refinement during the third week of life that is powerfully controlled by visual experience. Given that the ccc regulates phases of retinogeniculate refinement that culminate prior to this period, until recently, whether and how cytokines contribute to this late phase of development had yet to be resolved. Because it was previously observed that the phagocytic activity of microglia decreases drastically around P7, a prevailing view was that microglia do not participate in this late phase of sensory-dependent refinement in the dLGN.

This view was recently challenged by the discovery of a TNF family cytokine pathway that controls late-stage retinogeniculate refinement. In an *in vivo* single-cell RNA-sequencing screen for genes expressed in dLGN neurons in response to visual experience during this late stage of development, Cheadle et al. (67) discovered that visually stimulated neurons strongly increase the expression of the gene encoding the TNF receptor superfamily member Fibroblast Growth Factor-inducible protein 14 kDa (Fn14, also known as TNF receptor superfamily member 12a) (67). Intriguingly, visual stimulation concurrently induces the expression of the ligand of Fn14, the TNF superfamily member TNF-associated Weak Inducer of Apoptosis (TWEAK), in a subset of microglia in the dLGN (32). While the roles of TWEAK-Fn14 signaling outside of the brain in processes such as muscular degeneration, cell proliferation, angiogenesis, and inflammation have been extensively characterized, virtually nothing was known about the roles of this pathway in the healthy brain (68).

Through a combination of electrophysiological, ultrastructural, and molecular approaches applied to the retinogeniculate circuit, the authors found that Fn14 functions when not bound by TWEAK to strengthen retinogeniculate connectivity by increasing the number of synapses. However, at retinogeniculate synapses near the subset of stimulated microglia that express TWEAK, microglia-derived TWEAK binds Fn14 to trigger the deconstruction of dendritic spines and ultimately remove functional synapses (32, 67). Notably, TWEAK and Fn14 are dispensable for early stages of retinogeniculate refinement including ESS which is coordinated by the ccc (17). Likewise, phagocytic engulfment of synapses occurs normally in mice lacking TWEAK or Fn14 across all time points. Thus, TWEAK-Fn14 signaling selectively coordinates a late-stage of sensory experience-dependent synapse refinement through a microglia-driven, non-phagocytic mechanism of synapse elimination (**Figure 2B**).

Whereas ccc and IL-33 signaling play varied roles across multiple neural circuits, until recently it was not known whether TWEAK-Fn14 signaling extends beyond the retinogeniculate synapse to remodel other circuits during development or in the adult, and whether this pathway may also be relevant to disease. A recent study by Nagy et al. (69) showed that exogenous application of recombinant TWEAK dampens long-term potentiation, a measure of activity-dependent synaptic strengthening, in the hippocampus. Moreover, suppression of endogenous TWEAK using blocking antibodies ameliorated Fn14-dependent synaptic deficits in mouse models of stroke and Alzheimer's disease, indicating not only that the TWEAK-Fn14 pathway is an important synaptic organizer across multiple circuits in the brain but also that disruptions in TWEAK-Fn14 signaling may contribute to neurological dysfunction related to brain disorders. Therapies focused on interfering with TWEAK-Fn14 signaling may therefore have potential for treating neurodegeneration and stroke-related illness.

### Fractalkine

While complement plays multifaceted roles in the hippocampi of mature mice, evidence linking complement to the development of hippocampal circuitry is lacking. Instead, research points to signaling between the neuronal chemokine Fractalkine (CX3CL1) and its microglia-expressed receptor, CX3CR1, as mediators of synapse development in the hippocampus. In contrast to the pathways thus far described, CX3CL1 and CX3CR1 are both highly enriched in the brain where they have been shown to play a wide variety of roles related to brain health and function [reviewed in (70)] including: the migration and colonization of microglia within the hippocampus (16), neuronal survival (71, 72), microglial morphology and motility (73), maturation of inhibitory and excitatory synapses (74), and functional neurotransmission (75–78). Many of these deficits arise within the first three weeks of life at a time when excess hippocampal synapses are undergoing activity-dependent pruning (79). Animals lacking CX3CR1 maintain an excess of hippocampal CA1 synapses exhibiting decreased synaptic strength compared to WT, suggesting a concurrent impairment in synapse elimination and an inability of synapses to properly mature in the absence of fractalkine signaling (16, 78). These deficits in synaptic connectivity persist into adulthood and are accompanied by reduced local field potentials and altered social interactions (80).

Similar to the ccc, fractalkine signaling is largely dispensable for visual cortex development *in vivo*, as visual acuity, experience-dependent synaptic potentiation, and ocular dominance plasticity are all normal in mice lacking CX3CR1 (81). Likewise, microglial morphology, density, motility, and interactions with synapses in V1 are also unchanged by loss of CX3CR1 (82). On the contrary, studies in somatosensory cortex have revealed a requirement of fractalkine signaling for the recruitment of microglia to "barrels", clusters of thalamocortical (TC) inputs that correspond to the territory selectively innervated by each whisker, between P5 and P7. CX3CR1 is also required for the functional maturation of TC connectivity, the pruning of excess TC inputs that occurs in response to sensory lesioning, and experience-dependent synaptic remodeling (33). Fractalkine KO mice and mice subjected to pharmacological inactivation of Adam10, a metalloprotease that cleaves CX3CL1 into a soluble form enabling it to bind its microglial receptor, both show similar deficits in synapse pruning and maturation as those observed in the CX3CR1 KO (**Figure 2E**). Notably, this study found C1q to be dispensable for synapse development in somatosensory cortex, highlighting that different immune pathways are specialized to regulate distinct circuits (33). Altogether, these detailed studies across a multitude of circuits in the brain show that activity-dependent processes are controlled by microglia and cytokine signaling.

## Activity-Dependent Expression of Cytokines in the Brain

Now that it is becoming clear that activity shapes microglial and cytokine function, it is important to disentangle the mechanisms underlying this process. Emerging data suggest that one way in which activity influences cytokine function is through tight control of cytokine and cytokine receptor expression. Supporting this possibility, signaling between TWEAK and Fn14, a cytokine pathway through which microglia remove developing synapses in the dLGN as described above, is coordinated by the concurrent transcriptional induction of each signaling partner in microglia and neurons, respectively, in response to visual stimulation. The result that microglia induce TWEAK expression in response to experience was remarkable because, while neurons have been known to mount transcriptional responses to sensory cues for decades, it was not expected that an immune population such as microglia may also respond at such a fundamental level to sensory information from the external world. An interesting feature of the sensory-dependent induction of TWEAK expression in microglia is its restriction to a subset of microglia, which suggests heterogeneity in the responses of microglia to a given stimulus and indicates that different microglia within the same tissue may perform different functions depending upon the cytokines that they express. Consistent with such functional heterogeneity, retinogeniculate synapses nearby microglia that induce TWEAK are more likely to be eliminated than retinogeniculate synapses near microglia that do not induce TWEAK. In contrast to TWEAK expression in microglia, Fn14 induction occurred across *all* excitatory neurons in the dLGN, suggesting that at least in this context neuronal responses to experience are more stereotyped and synchronous than the responses of microglia. The difference in gene induction is not unexpected, as neurons are directly activated by synaptic transmission while microglia are likely to be activated through less direct pathways, such as molecular signals from active neurons. Overall, these data highlight that the transcriptional control of cytokine and cytokine receptor expression across different cell types is one way in which activity determines cytokine function.

These studies of TWEAK and Fn14 indicate that sensory stimuli can induce the cell-type-specific expression of cytokine signaling partners to coordinate precise interactions between distinct cell types (32). Intriguingly, further analysis of the single-cell transcriptomic dataset profiling experience-dependent genes in dLGN neurons showed that Fn14 is not the only immune signaling molecule whose neuronal expression depends upon activity. For example, Fractalkine (CX3CL1) is also highly induced in neurons (the same neurons that induce Fn14) in response to visual stimulation, suggesting that fractalkine signaling from neurons to microglia is another circuit-specific example of experience controlling cytokine function. Yet another example of experience-dependent neuron to microglia cytokine signaling is found in the hippocampus, where increased sensory stimulation through EE induces IL-33 expression in neurons which binds its Il1rl1 receptor in microglia to engage microglia in synaptic plasticity (66). Thus, inducible transcription of cytokines is one method through which heightened neural activity can precisely control cytokine signaling from microglia to neurons, and vice versa.

In addition to the direct transcriptional control of cytokine and cytokine receptor expression, neural activity may also mediate aspects of cytokine signaling between partners whose expression is activity-*independent*. An excellent example of this is found in the developing somatosensory cortex, where Gunner et al. (33) discovered that, although CX3CL1 and CX3CR1 expression does not require experience, fractalkine signaling is controlled by experience via the induction of the expression of Adam10 in layer 4 neurons. Adam10 is a metalloprotease that, among other functions, cleaves neuronal CX3CL1 into its soluble form. Upon cleavage, soluble CX3CL1 binds CX3CR1 in neighboring microglia to induce synaptic pruning of thalamocortical inputs through phagocytic engulfment. This process thereby anatomically segregates distinct "barrel" regions of cortex innervated by independent whiskers (33). This intriguing finding opens up a host of other possible methods through which activity may coordinate specialized functions of cytokine signaling beyond direct transcriptional control of the signaling components themselves. For example, it will be interesting to determine whether the initiation of local protein translation or the posttranslational modification of microglial proteins are also regulated by activity.

## Discussion: Broader Impacts and New Horizons

It is now evident that cytokines are pleiotropic molecules that are repurposed throughout the CNS to mediate synapse development, plasticity, and function. However, the signals that maintain the specialized roles of microglia and cytokines in the brain are still being elucidated. Thus far, we reviewed evidence that neural activity is a brain-specific influence on immune signaling pathways that, to a large extent, determines their specialized functions in the CNS. Not only are microglial Ca^2+^ dynamics, motility, and interactions with synapses tightly and bimodally attuned to both increases and decreases in activity, microglia and cytokines also play integral roles in developmental and homeostatic processes that are activity-dependent. Although these data strongly suggest an important link between activity and microglial/cytokine function, the direct relationship between these two phenomena is not yet well-established.

Given that microglia are non-neuronal immune cells that lack intrinsic excitability and are not connected to sensory structures or neurons at the synaptic level, how microglia initially detect changes in neuronal activity is a question under active investigation. One possible mechanism through which microglia may respond to sensory stimulation is purinergic signaling. Upon stimulation, neurons release purine nucleotides such as adenosine and ATP which can act as signals to other cells such as microglia. The purinergic Gi coupled receptor, P2y12, in conjunction with the potassium channel TWIK-related halothane-inhibited K+ channel (THIK-1), has been shown to play critical roles in the recruitment of microglia to areas of high activity, and deletion or blunt inhibition of either has been shown to increase overall neural activity and seizure susceptibility, making P2y12/THIK-1 an ideal candidate for mediating interactions between activity and microglia (25, 34). Alongside P2y12/THIK-1's role in motility and suppression of neural activity, evidence suggests that activation of the P2y12/THIK-1 can induce the expression of inflammatory cytokine IL-1β and the initiation of NLRP3 inflammasomes (83). Therefore, P2y12/THIK-1 signaling is a strong candidate pathway for initiation of cytokine release by microglia upon increases in neural activity, and may prime microglia to remodel or remove synapses via structural mechanisms like phagocytic engulfment and/or contact-independent mechanisms like TWEAK-Fn14 signaling.

Yet, it should be noted that this P2y12/THIK-1 signaling is likely not the only pathway through which microglia both sense and induce large-scale synaptic changes. For instance, microglia are capable of directly monitoring levels of neurotransmitters and neuromodulators through canonical neurotransmitter receptors that microglia have been shown to express (84). Therefore, the intracellular signaling cascades these neurotransmitter receptors are coupled to may dictate which cytokines the microglial cell engages to remodel neural connectivity. These mechanisms of direct response to neurotransmitter release are consistent with the observation that microglia are highly motile cells that constantly sample synapses across the brain parenchyma. Another possibility is that heightened activity in neurons leads to the transcription, translation, secretion, and/or subcellular localization of non-neurotransmitter molecular signals capable of binding receptors on microglia. When such binding occurs, microglia may respond through intracellular signaling events leading to a global change in transcriptional profile. Finally, because changes in neurovascular blood flow, structural complexity, and BBB integrity accompany changes in neural activity, molecular signals from the blood or from the BBB itself (comprised of endothelial cells, pericytes, and astrocytes) may also inform microglia of changes in activity and recruit them to remodel synapses in response (85, 86). Determining how microglia sense changes in activity is therefore an important next step in defining the specialized roles of cytokine signaling in the brain.

As described above, transcriptional upregulation of cytokines and their receptors in both microglia and neurons has emerged as one point of control through which activity mediates cytokine function. While much is known about the gene programs and transcriptional mechanisms that coordinate activity-dependent gene expression in neurons, much less is known about this process in microglia. In addition to candidate-based approaches studying TWEAK-Fn14 and IL-33 signaling, a recent study investigated the gene programs induced in microglia of the striatum following DREADD-mediated activation of neighboring neurons. This approach identified multiple genes that are upregulated or downregulated in microglia following neuronal activation, including those that encode mediators of chemotaxis and actin polymerization being upregulated and those encoding baseline aspects of motility being downregulated (25). With the growing number of approaches for analyzing transcriptomic and genomic changes in individual cell types, the field is now poised to systematically define the gene programs within neurons, microglia, and other non-neuronal cells that are induced by activity. Moving forward, it will be important to assess these changes not only in response to artificial manipulation of neural activity such as through chemogenetic, optogenetic, and pharmacological methods but also in response to more naturalistic cues such as visual stimuli and EE. It will also be critical to analyze the epigenomic changes, such as chromatin accessibility and histone modification, that underlie the transcriptional responses of microglia to activity.

The evidence reviewed here suggests that activity not only influences microglial and cytokine function, but that cytokines and microglia provide feedback to neurons that sculpts activity. Thus, communication between activity and immune signaling mechanisms in the brain is a two-way street. For example, upon the transcriptional upregulation of TWEAK in microglia in response to experience, TWEAK binds Fn14 at synapses to remove them, thereby dampening neural activity in the dLGN overall. Moving forward, it will be important to understand the specific changes that cytokines elicit at synapses in order to shape activity, a line of investigation that has the potential to reveal roles for cytokines not only in local circuit homeostasis but also computational aspects of brain function including sensory integration and motor coordination. This research direction is important for building upon studies elucidating the roles of microglia and cytokines in organizing synapses locally by assessing whether microglia can detect differences in activity patterns driven by selective features of neural circuit function.

Finally, as mounting evidence suggests that immune dysfunction contributes to neurological diseases arising across the lifespan, the implications of cytokine function in the brain have taken on a new translational urgency. For example, neurological diseases that are highly correlated with neuroinflammation and cytokine upregulation include Alzheimer's disease (AD), multiple sclerosis (MS), and stroke. In a widely used mouse model of Alzheimer's disease, C1q is deposited upon hippocampal synapses before the onset of disease progression. This leads to the complement-mediated, inappropriate phagocytosis of functional synapses by microglia resulting in disease-linked synapse loss and cognitive decline (50). Similarly, inhibiting TWEAK-Fn14 signaling in an AD mouse model ameliorated deficits in synapse plasticity in the hippocampus, suggesting that multiple immune pathways can contribute to the same pathological conditions that affect neural connectivity (69). In a mouse model of MS, where synapse loss in grey matter regions precedes axonal degeneration (independent of demyelination) (87), genetic deletion of C3 can ameliorate both microglial activation as well as neural dysfunction (88) suggesting that targeting the complement system may have therapeutic potential for treating chronic neuroinflammatory illnesses before symptoms arise. The therapeutic potential of targeting cytokines is not restricted to diseases of chronic neuroinflammation, but likely extends to acute stroke models as well, given that targeting TNFs (89) or ILs (90) ameliorates synaptic deficits in these conditions. Therefore, as extensively reviewed elsewhere (91–94) it is evident that therapeutically targeting cytokines may attenuate the deleterious effects of either acute or chronic insults characterized by increased cytokine load and subsequent neuroinflammation.

Extending beyond diseases such as stroke, MS, and AD, several lines of evidence also implicate microglia and cytokines in neurodevelopmental disorders like autism. For example, a recent study comparing the transcriptomes of individual cells from the brains of subjects with autism against non-autistic individuals identified microglia as the most impacted cell type at the transcriptomic level (95). In combination with the association of genetic mutations in neurodevelopmental disorders such as autism and schizophrenia within and near immune-related genes, most notably the MHC locus (96), such transcriptional studies support a role for immune signaling in these disorders. As the fundamental basis of microglial and cytokine function in the healthy brain is uncovered, it will be important to extend studies to mouse models of human disease and to human tissue in order to understand how cytokines may be aberrantly employed in the unhealthy brain to unravel neural wiring and derail neurological function. A better understanding of the relationship between cytokines and neural activity has the potential to not only expand what is known about the molecular basis of brain function in health but also to reveal core mechanisms of human neurological disease.

## Author Contributions

LC, AF, and YA wrote and edited the manuscript. All authors contributed to the article and approved the submitted version.

## Funding

Related work in the Cheadle lab is supported by the National Institute of Mental Health (NIMH) of the National Institutes of Health under award number 4R00MH120051-04.

## Conflict of Interest

The authors declare that the research was conducted in the absence of any commercial or financial relationships that could be construed as a potential conflict of interest.
